# Examining the Long-term Spillover Effects of a Pay-for-Performance Program in a Healthcare System That Lacks Referral Arrangements

**DOI:** 10.34172/ijhpm.2023.7571

**Published:** 2023-10-09

**Authors:** Chi-Chen Chen, Kuo-Liong Chien, Shou-Hsia Cheng

**Affiliations:** ^1^Department of Public Health, College of Medicine, Fu-Jen Catholic University, Taipei, Taiwan.; ^2^Institute of Epidemiology and Preventive Medicine, College of Public Health, National Taiwan University, Taipei, Taiwan.; ^3^Department of Internal Medicine, National Taiwan University, Taipei, Taiwan.; ^4^Population Health Research Center, National Taiwan University, Taipei, Taiwan.; ^5^Institute of Health Policy and Management, College of Public Health, National Taiwan University, Taipei, Taiwan.

**Keywords:** Pay-for-Performance, Spillover Effect, Multitasking, Diabetes Mellitus, Intermediate Clinical Outcome

## Abstract

**Background:** Several studies have examined the intended effects of pay-for-performance (P4P) programs, yet little is known about the unintended spillover effects of such programs on intermediate clinical outcomes. This study examines the long-term spillover effects of a P4P program for diabetes care.

**Methods:** This study uses a nationwide population-based natural experimental design with a 3-year follow-up period under Taiwan’s universal coverage healthcare system. The intervention group consisted of 7688 patients who enrolled in the P4P program for diabetes care in 2017 and continuously participated in the program for three years. The comparison group was selected by propensity score matching (PSM) from patients seen by the same group of physicians. Each patient had four records: one pertaining to one year before the index date of the P4P program and the other three pertaining to follow-ups spanning over the next three years. Generalized estimating equations (GEEs) with difference-in-differences (DID) estimations were used to consider the correlation between repeated observations for the same patients and patients within the same matched pairs.

**Results:** Patients enrolled in the P4P program showed improvements in incentivized intermediate clinical outcomes that persisted over three years, including proper control of glycated hemoglobin (HbA1c) and low-density lipoprotein cholesterol (LDL-C). We found a slight positive spillover effect of the P4P program on the control of non-incentivized triglyceride [TG]). However, we found no such effects on the non-incentivized high-density lipoprotein cholesterol (HDL-C) control.

**Conclusion:** The P4P program has achieved its primary goal of improving the incentivized intermediate clinical outcomes. The commonality in production among a set of activities is crucial for generating the spillover effects of an incentive program.

## Background

**Key Messages**

**Implications for policy makers**

- This study suggests that the pay-for-performance (P4P) program for diabetes care has achieved its primary objective of improving incentivized intermediate clinical outcomes such as glycated hemoglobin (HbA1c) and low-density lipoprotein cholesterol (LDL-C).
- It also suggests that a non-incentivized measure (triglycerides, TGs) was also improved owing to the “commonality in procedure” phenomenon.
- Policy-makers may need to evaluate the spillover effects of a financial incentive program on non-targeted conditions and non-incentivized indicators in various healthcare settings.

**Implications for the public**

Several studies have investigated the intended effects of pay-for-performance (P4P) programs in various health systems, yet little is known about the unintended spillover effects of such programs. The single-payer Taiwanese universal health scheme launched a P4P program for diabetes care in 2001. This study demonstrates that the P4P program significantly improves incentivized intermediate clinical outcome measures, such as glycated hemoglobin (HbA1c) and low-density lipoprotein cholesterol (LDL-C), and the effect persist over three years. In addition, the results of this study suggest a positive spillover effect of the P4P program on non-incentivized triglyceride (TG) control. These findings support the phenomenon of commonality in production among a set of activities crucial for generating spillover effects of an incentive program.

Over the past two decades, an increasing number of pay-for-performance (P4P) programs, which use financial incentives to better motivate physicians and improve the quality of healthcare, have been adopted worldwide.^1-5^ A P4P program is expected to enhance the quality of care by offering incentives to providers. Financial incentives reward certain services based on pre-determined benchmarks. These programs generally provide incentives to healthcare providers to improve the process of care (eg, checking the glycated hemoglobin [HbA1c] levels of patients with diabetes) and intermediate outcomes (eg, controlling the HbA1c levels of patients with diabetes). The measures are based on the recommendations of clinical practice guidelines. Good adherence to clinical guidelines can improve health outcomes for patients. Therefore, considering the incentives offered under P4P programs, healthcare providers might devote more effort to specific conditions or indicators that are rewarded and pursue better care outcomes for patients.^1^

 However, P4P programs may have potential unintended consequences,^1,2,4^ such as spillover effects on neglecting conditions or activities that are not directly rewarded.^4^ Holmstrom and Milgrom proposed the “multitasking problem” to illustrate the possibility of spillover effects.^6^ In a P4P scheme, healthcare providers might pay more attention to indicators tied to financial incentives and neglect those that are not incentivized. Therefore, P4P programs may improve the performance of incentivized items at the expense of the unfavorable performance of non-incentivized items, resulting in a decline in the quality of care for patients. This phenomenon is called a “negative spillover effect.”

 The problem of multitasking might be alleviated if the activities undertaken in a process are similar or co-occur, because certain conditions or indicators rely on similar inputs.^7,8^ This means that, under a P4P program, non-incentivized activities may be indirectly rewarded when they share commonalities with incentivized activities. This phenomenon is called a “positive spillover effect” (or halo effect). Therefore, commonalities in production among a set of activities are crucial for generating positive or negative spillover effects from an incentive program.

 Although the intended effects of P4P programs have been well documented,^1-5^ little is known about their spillover effects, and the results tend to be inconclusive. Some studies have found that P4P programs have a positive spillover effect on non-incentivized conditions or indicators,^9-12^ whereas others have found negative spillover effects, because of the possible neglect of non-incentivized aspects of care by healthcare providers over the long term.^13,14^ Some studies have found no spillover effect at all of P4P programs.^8,15^ The aforementioned discrepancies in the findings might be attributed to variations in the types of P4P programs implemented, the quality of the methodology used, and differences in the research setting.

 Most empirical studies on the spillover effects of P4P programs have been performed in the United States^8,11,15^ or the United Kingdom.^9,10,13,14^ The spillover effects of P4P programs in the United States have tended to be small, which might be attributable to the diluting effect of the small incentive size based on multiple payers and the phenomenon in which payers often consider only a fraction of the targeted healthcare providers.^2,3^ The P4P programs in the United Kingdom have been implemented nationally. Most of these programs are designed for primary care settings and reward general practitioners based on the quality-of-service delivery. However, healthcare systems in many Asian countries such as Japan, Korea, and Taiwan lack general practitioners (as gatekeepers) or formal referral mechanisms and are mainly specialist-based care.

## Methods

 This study used a natural experimental design with population-based longitudinal data to examine the long-term spillover effects of a P4P program. The analysis was based on claims data for healthcare utilization from 2016 to 2020 in Taiwan.

###  Pay-for-Performance Program for Diabetes Care

 Since the end of 2001, Taiwan’s single-payer, the National Health Insurance Administration (NHIA), has implemented nationwide P4P programs for several chronic conditions. For the P4P program for diabetes care, physicians who specialize in metabolic disorders or endocrinology or those who have completed training for the diabetes shared care program can voluntarily participate in the P4P program for diabetes care. Participating physicians can recruit patients for the program. The P4P program pays participating physicians three fees: the P4P management fee for the initial enrollment visit, an extra fee for a comprehensive follow-up visit, and an annual evaluation fee. The required and recommended services include a medical history examination, physical examination (for example, ophthalmoscopic or foot examination), laboratory evaluation (for example, HbA1c check or cholesterol checks), management plan, and diabetes self-management education, which are clearly defined in the P4P program for diabetes care.^16^

 Outcome-based quality indicators have been gradually incorporated into the P4P program for diabetes care. Between 2001 and 2006, the financial incentives of the diabetes P4P program focused only on participation or process-based care (eg, performing HbA1c or cholesterol checks). In late 2006, the NHIA implemented a new strategy to better reward the health outcomes by paying an additional bonus for improvement in intermediate clinical outcome measures. A composite score was developed for each participating physician based on measures including the complete follow-up visit rate and control of HbA1c and low-density lipoprotein cholesterol (LDL-C) levels. Under this scheme, physicians receive an extra outcome-based reward (NT$ 1000 or US$ 33 per patient) if their composite scores rank among the top 25% of participating physicians. This plan is called the “pay for excellence” incentive. In 2009, the NHIA introduced a “pay-for-improvement” incentive to encourage physicians to focus on improving the composite scores of patients. Physicians receive an extra reward (NT$ 500 or US$ 17 per patient) if the composite score of a patient improves or is maintained over two years.^16^ In short, both process-based (eg, HbA1c check) and intermediate clinical outcome-based indicators (eg, proper control of HbA1c) for the recommended examination and laboratory tests have been incorporated into the diabetes P4P program. However, not all the intermediate clinical outcome indicators of the examinations/tests are incentivized in the program.^16^

###  Participants

 Adult patients (aged 20 or older) diagnosed with type-2 diabetes were identified using the International Statistical Classification of Diseases and Related Health Problems 10th Revision (ICD-10) code E11. Because clinical data for intermediate outcomes (eg, HbA1c level) became available starting in 2015, we included only patients with at least three diabetes-related physician visits and laboratory test results every year from 2016 to 2020. Patients with diabetes who were enrolled in the nationwide P4P program in 2017 were included in the intervention group. The index date for each patient was defined as the date of enrollment in the P4P program between January 1, 2017 and December 31, 2017. The study included only participants who remained in the program throughout the observation period from 2017 to 2020.

 Selection bias cannot be ruled out because the patients enrolled in the program were purposively selected by their physicians and voluntarily enrolled in the P4P program.^17,18^ To minimize the potential impact of selection bias, we adopted a two-step sample selection: identifying patients visiting the same group of physicians and using the propensity score matching (PSM) approach to select patients. In the first step, we identified the most frequently visited physicians for the diabetes care of the 9119 patients enrolled in the P4P program. All diabetic patients who visited the shortlisted set of physicians were identified. Patients who had never been enrolled in the P4P program were considered suitable for the comparison group. A total of 2649 physicians were consulted by 9119 patients (pre-matched) in the intervention group, and 21 651 patients (pre-matched) were selected via PSM in the comparison group.

 Subsequently, in the second step, we used a PSM approach to select appropriate patients to form the comparison group.^19^ For each patient, we created a propensity score that estimated the probability of their enrollment in the P4P program based on their characteristics using a generalized estimating equations (GEEs) model considering the effects of patient clustering among particular physicians.^20^ The characteristics of patients considered in the matching process included their age, sex, Charlson comorbidity index (CCI) score,^21^ diabetes complication severity index (DCSI) score,^22^ likelihood of hospitalization in the previous year, and baseline HbA1c values. The characteristics of healthcare providers included in the model were the accreditation level^23^ and location of the hospital or community clinic that was most frequently visited by a given patient.

 Based on the propensity scores, we used the caliper matching method with 1:2 matching between the intervention and comparison groups. Finally, 7688 patients (post-matched) were enrolled from the P4P program, and the PSM process yielded 15 376 patients (post-matched) in the comparison group. In addition, we calculated absolute standardized differences in baseline characteristics between the intervention and comparison groups. Standardized differences less than 10% indicated acceptable matching.^24-26^

 The observation period ranged from one year before the index date in 2017 to three years of subsequent follow-up. As the subjects in the comparison group did not have an index date unlike those enrolled in the P4P program, they were assigned a pseudo-index date of their matched counterparts in the intervention group. In all, 23 064 patients and 92 256 patient years were included in the analysis. The unit of analysis was the number of patient-years.

###  Measurement of Variables

 In terms of dependent variables, four intermediate clinical outcome indicators were included in this study: two incentivized and two non-incentivized indicators under the P4P scheme. In the analysis, the two incentivized indicators measured whether the patient had proper control of their HbA1c (HbA1c <7%) and LDL-C levels (LDL-C <100 mg/dL) during the study period. High-density lipoprotein cholesterol (HDL-C) and triglyceride (TG) levels are also crucial for the management of dyslipidemia in patients with diabetes.^27^ Therefore, we included two non-incentivized indicators to measure whether the patient had proper control over their HDL-C (HDL-C >40 mg/dL) and TG levels (TGs <200 mg/dL).

 The leading independent variables were patient enrollment in the P4P program, time dummy variables for the three years after the index date, and three interaction terms for the previously described variables. The following covariates were controlled in the regression models: patient characteristics (sex, age, CCI score, DCSI score, and likelihood of hospitalization in the previous year) and healthcare provider characteristics (accreditation level and location).

###  Statistical Analyses

 To examine the spillover effects of the P4P program, we fitted the GEEs with difference-in-differences (DID) estimation to longitudinal data that considered the correlation between repeated observations for the same patients and patients within the same matched pairs as well as considered the unobserved time-invariant characteristics for patients.^20^ The likelihood of the patient having proper control on the four intermediate clinical outcome indicators was analyzed using a logit link function and had a binominal distribution. The specifications are as follows:

*logit Y*_it_* = β*_0_ + *β*_1_ × *P*4*P*_it_* + β*_2_ × *Year*1_it_* + β*_3_ × *Year*2_it_* + β*_4_ × *Year*3_it_* + β*_5_ × *P*4*P*_it_ × *Year*1_it_ + *β*_6_* × P*4*P*_it_ × *Year*2_it_ + *β*_7_ × *P*4*P*_it_* × Year*3_it_ + *β*_8_ × *X*_it_ + *β*_9_ × *Z*_i_ + *ε*_it_

 where *Y*_it_ is the likelihood of good control on an intermediate clinical outcome for patient*i*during period* t. *The *P*4*P*_it _dummy indicates whether the patient was enrolled in the P4P program. *Year*1_it_, *Year*2_it_, and *Year*3_it_ are dummy variables that indicate observations for over 3 years after the index date of the P4P program. The DID estimates of the effects of the P4P program are captured by the coefficients *β*_5_, *β*_6_, and *β*_7_ on the three interaction terms *P*4*P*_it_* × Year*1_it_, *P*4*P*_it_* × Year*2_it_, and *P*4*P*_it_* × Year*3_it_, respectively. Parameters *X*_it_ and *Z*_i_ represent a set of variables that measure time-variant and time-invariant covariates (participant’s sex). All statistical analyses were performed using the SAS version 9.4 (SAS Institute) and Stata version 15.1 (StataCorp).

## Results

###  Descriptive Analysis

Table 1 lists the baseline characteristics of the pre- and post-matched participants in the intervention and comparison groups. In the pre-matched sample, patients diagnosed with diabetes in the intervention group were younger, had higher DCSI and CCI scores, and controlled their baseline HbA1c levels less well. Furthermore, they had a higher likelihood of hospitalization in the previous year and tended to receive care at a medical center/regional hospital. The PSM process resulted in a more balanced distribution of the characteristics of patients and their care providers in the intervention and comparison groups. All absolute standardized differences were less than 10%, indicating acceptable matching results.

**Table 1:** Characteristics of Patients With Diabetes in the Pre- and Post-matched Samples at Baseline

**Characteristics**	**Pre-matched Sample**					**Post-matched Sample**				
	**Intervention Group**		**Comparison Group**			**Intervention Group**		**Comparison Group**		
	**No.**	**%**	**No.**	**%**	**Absolute Standardized Differences**	**No.**	**%**	**No.**	**%**	**Absolute Standardized Differences**
All	9119		21651			7688		15376		
Gender										
Female	4338	47.57	9949	45.95	0.03	3525	45.85	7079	46.04	0.00
Male	4781	52.43	11 702	54.05	0.03	4163	54.15	8297	53.96	0.00
Age group										
<60	3523	38.63	7746	35.78	0.06	3029	39.40	5576	36.26	0.06
60-74	4351	47.71	9740	44.99	0.05	3446	44.82	7349	47.8	0.06
75+	1245	13.65	4165	19.24	0.15	1213	15.78	2451	15.94	0.00
DCSI score										
Score 0	3139	34.42	8249	38.10	0.08	2897	37.68	5544	36.06	0.03
Score 1	2092	22.94	5047	23.31	0.01	1818	23.65	3637	23.65	0.00
Score 2+	3888	42.64	8355	38.59	0.08	2973	38.67	6195	40.29	0.03
CCI score										
Score 1	4463	48.94	11022	50.91	0.04	3814	49.61	7767	50.51	0.02
Score 2	3721	40.8	8535	39.42	0.03	3073	39.97	6193	40.28	0.01
Score 3+	935	10.25	2094	9.67	0.02	801	10.42	1416	9.21	0.04
Hospitalization in the previous year										
No	7374	80.86	18 481	85.36	0.12	6494	84.47	13 088	85.12	0.02
Yes	1745	19.14	3170	14.64	0.12	1194	15.53	2288	14.88	0.02
Control of HbA1c at baseline period										
Poor control (HbA1c ≥7)	5724	62.77	11 182	51.65	0.23	4337	56.41	8449	54.95	0.03
Better control (HbA1c <7)	3395	37.23	10 469	48.35	0.23	3351	43.59	6927	45.05	0.03
Mean value of HbA1c at baseline (mean SD)	7.65	1.38	7.28	1.21	0.29	7.35	1.08	7.29	1.09	0.06
Accreditation level										
Medical center/regional hospital	3766	41.3	7045	32.54	0.18	2938	38.22	5368	34.91	0.07
District hospital/clinics	5353	58.7	14 606	67.46	0.18	4750	61.78	10 008	65.09	0.07
Location of hospitals										
Taipei and northern regions	4258	46.69	11 910	55.01	0.17	4045	52.61	8072	52.5	0.00
Central and southern regions	2820	30.92	5810	26.83	0.09	2227	28.97	4425	28.78	0.00
Kao-ping and eastern regions	2041	22.38	3931	18.16	0.11	1416	18.42	2879	18.72	0.01

Abbreviations: DCSI, Diabetes Complication Severity Index; CCI, Charlson Comorbidity Index; SD, standard deviation; HbA1c, glycated hemoglobin.

 Figure presents the changes in the outcome measures over the study period. For the incentivized indicators, proper control of HbA1c levels was lower in the intervention group than in the comparison group in the baseline year (43.59% vs. 45.05%). After the first year of the P4P program, the rate of proper control of HbA1c increased in the intervention group (49.08%) and was higher than that in the comparison group (46.85%). For proper control of LDL-C, the rates in the intervention and comparison groups increased after the intervention (ranging from 56.18% to 70.51% in the intervention group; 57.30% to 66.77% in the comparison group). For non-incentivized indicators, we found that the rates of proper control on HDL-C steadily increased in both the intervention and comparison groups over four years. Finally, we found a noticeable increase in the rate of proper control of TG levels in the intervention group after enrollment, compared with a slight increase in the comparison group.

**Figure:**
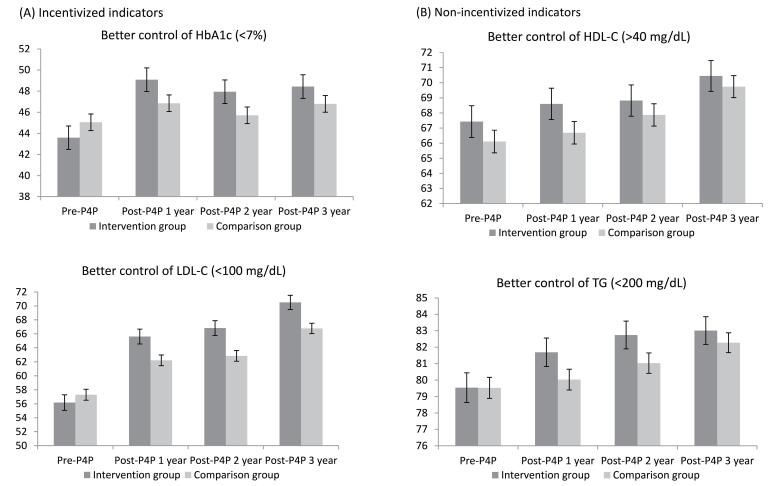
Distribution of Intermediate Clinical Outcome Measures by Intervention Status Over Study Period. Abbreviations: P4P, pay-for-performance; HbA1c, glycated hemoglobin; HDL-C, high-density lipoprotein cholesterol; LDL-C, low-density lipoprotein cholesterol; TG, triglyceride.

###  Results From Regression Models

Table 2 lists the results obtained from the DID estimates in the GEEs model. For the incentivized indicator HbA1c, patients enrolled in the P4P program were more likely than their counterparts to have proper control of their HbA1c levels during the post-P4P period. The odd ratios (ORs) of the interaction terms were positive and significant from the first to third years after the intervention (OR was 1.166 in the first year [95% confidence interval [CI]: 1.106-1.230], 1.168 in the second year [95% CI: 1.103-1.236], and 1.138 in the third year [95% CI: 1.073-1.206]). In respect of the proper control of LDL-C, we also found that the DID parameters were positive and significant, with OR being 1.199 (95% CI: 1.128-1.273), 1.232 (95% CI: 1.155-1.314), and 1.231 (95% CI: 1.150-1.318) for the three years after the intervention.

**Table 2:** Adjusted Estimations of the Effects of the Pay-for-Performance Program on the Incentivized and Non-incentivized Intermediate Clinical Outcomes

Characteristics	**Incentivized Items**						**Non-incentivized Items**					
	**HbA1c (<7%)**			**LDL-C (<100 mg/dL)**			**HDL-C (>40 mg/dL)**			**TGs (<200 mg/dL)**		
	**OR**	**95% CI**		**OR**	**95% CI**		**OR**	**95% CI**		**OR**	**95% CI**	
P4P group (reference group: comparison group)	0.948	0.897	1.002	0.970	0.918	1.026	1.071	1.009	1.138	1.018	0.951	1.090
Period (reference group: pre-P4P period)												
Post-P4P 1 year	1.069	1.038	1.100	1.208	1.169	1.247	1.030	1.001	1.059	1.014	0.978	1.052
Post-P4P 2 year	1.010	0.979	1.042	1.225	1.182	1.269	1.090	1.058	1.123	1.063	1.023	1.105
Post-P4P 3 year	1.047	1.013	1.081	1.436	1.384	1.491	1.198	1.161	1.236	1.138	1.094	1.185
Interaction term between P4P group and period (reference group: comparison group in pre-P4P period)										
P4P group×Post-P4P 1 year	1.166	1.106	1.230	1.199	1.128	1.273	1.036	0.985	1.089	1.114	1.044	1.189
P4P group×Post-P4P 2 year	1.168	1.103	1.236	1.232	1.155	1.314	0.986	0.936	1.040	1.122	1.047	1.202
P4P group×Post-P4P 3 year	1.138	1.073	1.206	1.231	1.150	1.318	0.974	0.922	1.029	1.051	0.980	1.128
Male	0.880	0.843	0.918	1.211	1.162	1.263	0.325	0.308	0.342	0.809	0.766	0.855
Age group (reference group: <60)												
60-74	1.354	1.300	1.410	1.239	1.189	1.292	1.196	1.147	1.247	1.557	1.482	1.635
75+	1.301	1.230	1.376	1.559	1.472	1.652	1.038	0.977	1.103	1.864	1.737	2.001
DCSI score (reference group: score 0)												
Score 1	0.946	0.912	0.981	1.189	1.142	1.238	0.950	0.916	0.986	0.974	0.931	1.019
Score 2	0.938	0.904	0.974	1.296	1.245	1.349	0.914	0.880	0.950	0.982	0.938	1.029
CCI score (reference group: score 1)												
Score 2	1.061	1.030	1.093	0.991	0.959	1.023	0.957	0.929	0.987	1.016	0.980	1.054
Score 3+	1.128	1.073	1.185	0.976	0.925	1.030	0.886	0.842	0.933	0.987	0.926	1.053
Hospitalization in the previous year (reference group: no)	1.078	1.046	1.112	1.016	0.982	1.052	0.967	0.938	0.997	0.965	0.928	1.004
Medical center/regional hospital (reference group: district hospital/clinics)	0.960	0.930	0.991	1.084	1.047	1.122	0.955	0.924	0.987	1.053	1.012	1.097
Location of hospitals (reference group: Kao-ping and eastern)												
Taipei and northern regions	0.725	0.686	0.767	0.831	0.786	0.878	1.001	0.938	1.069	0.850	0.791	0.913
Central and southern regions	0.841	0.792	0.894	0.970	0.913	1.030	1.018	0.948	1.093	0.967	0.893	1.047

Abbreviations: OR, odds ratio; CI, confidence interval; P4P, pay-for-performance; DCSI, Diabetes Complication Severity Index; CCI, Charlson Comorbidity Index; HbA1c, glycated hemoglobin; LDL-C, low-density lipoprotein cholesterol; HDL-C, high-density lipoprotein cholesterol; TGs, triglycerides.

 For the non-incentivized indicator TGs, the result was similar to that of the aforementioned incentivized indicators. Patients in the P4P program were more likely than their counterparts to have proper control of their TG levels in the first and second years after the P4P program (OR of 1.114 [95% CI: 1.044-1.189], 1.122 [95% CI: 1.047-1.202]). However, there was no significant effect in the third year after the P4P program (OR of 1.051 [95% CI: 0.980-1.128]). Conversely, we found no spillover effects of the P4P program on the non-incentivized HDL-C control in the three years after the P4P program (OR of 1.036 in the first year [95% CI: 0.985-1.089]; 0.986 in the second year [95% CI: 0.936-1.040]; and 0.974 in the third year [95% CI: 0.922-1.029]) (Table 2).

###  Sensitivity Analysis

 Sensitivity analyses were performed to validate the robustness of the findings. First, we used a continuous scale of the intermediate clinical outcome variables instead of a binary one. The analyses yielded results similar to those described earlier (Supplementary file 1, Table S1). Second, we performed analyses stratified by sex and found partly similar results (Table S2). Third, we performed analyses stratified by the status of multiple chronic conditions (<2 chronic conditions or ≥2 chronic conditions, excluding diabetes). In this study, the number of chronic conditions was measured using nine common chronic conditions that often require ongoing medication management, including hypertension, heart disease, cerebrovascular disease, pulmonary disease, chronic renal disease, arthritis/degenerative joint diseases, depression/anxiety-associated diseases, and cancer. Most of the results were similar to previous findings, except for patients with ≥2 chronic conditions, in terms of TG outcomes (Table S3). Finally, we used random-intercept models with DID estimation to consider unobserved time-invariant covariates and considered the correlation between repeated observations for the same patient. Instead of the odds ratios, we calculated the average marginal effects for all estimates and used bootstrapping with 100 replications to acquire standard errors (Table S4). Previously, a similar approach has been used in the econometric literature. These sensitivity analyses yielded results similar to our main results.

## Discussion

###  Effect of P4P Programs on Incentivized Outcomes

 The effect of a P4P program on intermediate clinical outcomes tended to be inconclusive in a previous systematic review.^5^ For example, under the Quality of Outcome Framework (QOF) in the United Kingdom, Vamos et al^28^ and Alshamsan et al^29^ performed an interrupted time series analysis and found that the trend in the proper control of HbA1c in patients with diabetes after the introduction of the QOF worsened relative to the pre-QOF trend. In contrast, we found that the P4P program for diabetes had a positive long-term impact on incentivized intermediate outcomes, including proper control of HbA1c and LDL-C levels in patients. Several possible explanations can explain this outcome. First, the universal QOF scheme is designed for primary care settings to reward general practitioners for achieving quality indicators. However, in Taiwan, P4P programs are designed with disease-specific incentives and rewards are given to specialists to achieve pre-determined targets. In other words, focusing on the management of diabetes care based on clinical guideline-recommended services (eg, medication management) could lead to better glycemic control. Second, the P4P scheme for diabetes in Taiwan provides multiple incentives. From 2001 to 2006, incentives were exclusively based on process-of-care outcomes (eg, receipt of HbA1c testing). Incentives targeting intermediate clinical outcomes (eg, proper control of HbA1c) were incorporated in 2006, and a bonus for improvement in intermediate clinical outcomes was introduced in 2009. Combining multiple incentive designs might better motivate the care providers toward improving intermediate clinical outcomes.^1^

###  Spillover Effect of P4P Programs on Non-incentivized Outcomes

 Previous studies have raised concerns about the unintended effects of P4P programs.^1,2,4^ However, research on the spillover effects of P4P programs on non-incentivized clinical outcomes is limited, and the findings tend to be inconclusive.^8-15^ This study focused on whether the intended improvement might spill over to other non-incentivized aspects of care, resulting in a decline in the overall quality of care among patients with diabetes. Under the P4P program for diabetes care in Taiwan, checks of patient TGs and HDL-C (a process of care) are incentivized, but control of patients’ TG and HDL-C levels (intermediate clinical outcomes) are not incentivized. For patients with diabetes, a high TG or low HDL-C level is associated with a higher risk of vascular complications.^30^ Therefore, controlling patients’ TG and HDL-C levels is essential for diabetes management, which in turn affects the overall health outcome. The findings from this study suggest that the P4P did not affect the control of non-incentivized HDL-C levels among the intervention group even three years after enrollment. However, we found that the P4P program had a positive spillover effect on TG control (another non-incentivized parameter) in the first and second years after the P4P program.

 The inconsistent findings of the spillover effects seem intriguing; however, when we consider “commonality in production,” these seem reasonable. In the P4P program, the multitasking problem can be mitigated when the procedures used by physicians to address the incentivized and non-incentivized indicators are similar.^7,8^ We observed that the improvement in TG control (a non-incentivized outcome) occurred because it was addressed by physicians in a manner similar to that of HbA1c control (an incentivized outcome). The recommendations of the American Diabetes Association emphasize that lifestyle interventions and glycemic control are both beneficial for improving TG control.^27^ For example, metformin is a first-line pharmacological agent used for glycemic control in type-2 diabetes, and evidence has shown that metformin can significantly decrease TG levels in patients with diabetes.^31^ In other words, for patients enrolled in the P4P program for diabetes care, physicians might regularly monitor their blood glucose levels and improve their glycemic control using medications such as metformin, which also improves the TG level. However, the improvement in HDL-C control was not affected by the use of similar treatments. The effects of metformin on HDL-C levels are less evident.^32,33^ Lifestyle interventions such as weight loss, exercise, and diet control are better ways to manage HDL-C levels.^27^ Therefore, our findings support the idea that commonalities among a set of activities are crucial for financial incentive programs to generate positive or no spillover effects.^8,12^

###  Limitations

 This study had some limitations. First, the participants were not randomly assigned to the intervention or comparison groups, which may have caused selection bias. In this study, we used two-step sample selection strategies to minimize selection bias: selecting patients from the same physician and using PSM. Although the PSM matching strategy may have increased the similarity of participants in the intervention and comparison groups at baseline, it does not ensure that the two groups were similar before the intervention. Owing to the lack of available intermediate clinical outcome data, we performed a placebo DID analysis using the participants’ diabetes-related hospitalizations as proxies to examine the parallel trend assumption before the intervention. During 2007 and 2016 (before the P4P intervention in 2017), the placebo DID demonstrated small and insignificant changes in hospitalization for diabetes-related conditions, which implied similar trends in the two groups before the intervention (Table S5).^34^ Finally, the participants in this study were representative of patients with at least three diabetes-related physician visits and laboratory test results, which might not represent all diabetes patients in Taiwan.

## Conclusion

 This is one of the first studies to evaluate the long-term spillover effects of a P4P program on intermediate clinical outcomes under a single-payer healthcare system. This study provides evidence showing that the P4P program has significantly improved incentivized outcome measures. In addition, the P4P program has a positive spillover effect on a non-incentivized outcomes when the related measure is treated in a manner similar to that of the incentivized indicators. However, this study found no spillover effect on non-incentivized outcomes when the related measure is not treated in a manner similar to that of incentivized indicators. We speculate that the problem of multitasking can be mitigated if a set of indicators is addressed using similar treatment measures. Therefore, we suggest that the incentive design of P4P programs should consider “commonality in procedure” to facilitate positive spillover effects and avoid negative spillover effects. Future investigations are necessary to examine the spillover effects of a financial incentive program with both “targeted vs. non-targeted conditions” and “incentivized vs. non-incentivized indicators” under various health-care settings and adopt a more extended follow-up.

## Ethical issues

 This study was approved by the Institutional Review Board of Fu Jen Catholic University (C106074).

## Competing interests

 Authors declare that they have no competing interests.

## Funding

 The study was supported by grants from the National Science and Technology Council (NSTC 112-2410-H-002 -039 -MY3) in Taiwan. The funding source had no role in the study.

## Supplementary files

Supplementary file 1 contains Tables S1-S5.
