# Supplementary material for: Examining the Long-term Spillover Effects of a Pay-for-Performance Program in a Healthcare System That Lacks Referral Arrangements
Source: Int J Health Policy Manag. 2023 Oct 9;12:7571. doi: 10.34172/ijhpm.2023.7571 (PMC10699817; doi:10.34172/ijhpm.2023.7571)
Supplement: Supplementary file 1 — contains Tables S1-S5. [file ijhpm-12-7571-s001.pdf]

Article title: Examining the Long-term Spillover Effects of a Pay-forPerformance Program in a Healthcare System That Lacks Referral Arrangements

Journal name: International Journal of Health Policy and Management (IJHPM)

Authors' information: Chi-Chen Chen[1], Kuo-Liong Chien[2,3,4], Shou-Hsia Cheng[4,5]*

[1]Department of Public Health, College of Medicine, Fu-Jen Catholic University, Taipei, Taiwan.

[2]Institute of Epidemiology and Preventive Medicine, College of Public Health, National Taiwan University, Taipei, Taiwan.

[3]Department of Internal Medicine, National Taiwan University, Taipei, Taiwan.

[4]Population Health Research Center, National Taiwan University, Taipei, Taiwan.

[5]Institute of Health Policy and Management, College of Public Health, National Taiwan University, Taipei, Taiwan.

***Correspondence to**: Shou-Hsia Cheng, Email: shcheng@ntu.edu.tw

Citation: Chen CC, Chien KL, Cheng SH. Examining the long-term spillover effects of a pay-for-performance program in a healthcare system that lacks referral arrangements. Int J Health Policy Manag. 2023;12:7571. doi:10.34172/ijhpm.2023.7571

Supplementary file 1

Table S1. Adjusted estimations of the effects of the P4P program on the incentivized and nonincentivized intermediate outcomes with a continuous variable

Characteristics	Incentivized items						Nonincentivized items					
	HbA1c (<7%)			LDL-C (<100 mg/dl)			HDL-C (>40 mg/dl)			TGs (<200 mg/dl)		
	β	SE	p-value	β	SE	p-value	β	SE	p-value	β	SE	p-value
Intercept	7.233	0.019	<0.001	101.602	0.429	<0.001	49.575	0.204	<0.001	161.808	1.961	<0.001
P4P group (reference group: comparison group)	0.056	0.015	<0.001	0.382	0.337	0.257	0.381	0.163	0.019	-1.096	1.531	0.474
Period (reference group: pre-P4P period)												
Post-P4P 1 year	-0.022	0.007	0.001	-3.045	0.178	<0.001	0.394	0.055	<0.001	-0.726	0.667	0.276
Post-P4P 2 year	0.013	0.007	0.062	-3.329	0.179	<0.001	0.754	0.055	<0.001	-2.567	0.670	<0.001
Post-P4P 3 year	-0.017	0.007	0.011	-5.329	0.180	<0.001	1.357	0.056	<0.001	-6.299	0.677	<0.001
Interaction term between P4P and period (ref: comparison group in pre-P4P period)												
P4P group×Post-P4P 1 year	-0.126	0.012	<0.001	-2.565	0.308	<0.001	0.374	0.095	<0.001	-8.047	1.153	<0.001
P4P group×Post-P4P 2 year	-0.137	0.012	<0.001	-3.073	0.308	<0.001	0.036	0.095	0.703	-5.583	1.153	<0.001
P4P group×Post-P4P 3 year	-0.110	0.012	<0.001	-3.101	0.308	<0.001	-0.020	0.095	0.834	-4.140	1.153	<0.001
Male	0.077	0.013	<0.001	-2.759	0.268	<0.001	-6.939	0.145	<0.001	8.036	1.298	<0.001
Age groups (reference group: <60)												
60-74	-0.166	0.010	<0.001	-2.610	0.240	<0.001	0.697	0.095	<0.001	-20.223	1.028	<0.001
75+	-0.166	0.015	<0.001	-5.816	0.335	<0.001	0.459	0.141	0.001	-26.300	1.472	<0.001
DCSI score (reference group: Score 0)												
Score 1	0.021	0.008	0.011	-1.763	0.212	<0.001	-0.157	0.072	0.029	0.783	0.838	0.350
Score 2	0.024	0.009	0.007	-2.895	0.217	<0.001	-0.242	0.076	0.002	-0.116	0.877	0.894
CCI score (reference group: score 1)												
Score 2	-0.025	0.007	<0.001	0.069	0.171	0.687	-0.049	0.058	0.398	0.523	0.677	0.440
Score 3+	-0.063	0.011	<0.001	-0.046	0.285	0.873	-0.292	0.097	0.003	1.236	1.132	0.275
Hospitalization in the previous year (reference group: No)	-0.044	0.007	<0.001	-0.331	0.175	0.058	-0.034	0.055	0.534	0.544	0.665	0.413
Medical center/regional hospital (reference group: District hospital/clinics)	0.010	0.008	0.189	-1.030	0.188	<0.001	-0.166	0.067	0.013	-0.381	0.762	0.617
Location of hospitals (reference group: Kao-ping and eastern regions)												
Taipei and northern regions	0.197	0.016	<0.001	2.974	0.346	<0.001	0.341	0.177	0.054	8.885	1.646	<0.001
Central and southern regions	0.078	0.018	<0.001	0.709	0.376	0.059	0.315	0.188	0.094	1.812	1.776	0.308

P4P—pay-for-performance program; DCSI—Diabetes Complication Severity Index; CCI—Charlson Comorbidity Index; HbA1c—*glycated hemoglobin;* LDL-C—*l*ow-density lipoprotein cholesterol; HDL-C—high-density lipoprotein cholesterol; TGs—triglycerides.

Table S2. Subgroup analysis for the sex groups

Characteristics	Incentivized items						Nonincentivized items					
	HbA1c (<7%)			LDL-C (<100 mg/dl)			HDL-C (>40 mg/dl)			TGs (<200 mg/dl)		
	ME	BSE	p-value	ME	BSE	p-value	ME	BSE	p value	ME	BSE	p value
**Female**												
Interaction term between P4P and period (reference group: comparison group in pre-P4P period)												
P4P group×Post-P4P 1 year	0.027	0.01	0.004	0.053	0.010	<0.001	0.006	0.00	0.302	0.013	0.007	0.055
P4P group×Post-P4P 2 year	0.021	0.01	0.028	0.050	0.011	<0.001	0.006	0.00	0.339	0.020	0.007	0.005
P4P group×Post-P4P 3 year	0.023	0.01	0.018	0.059	0.011	<0.001	0.005	0.00	0.400	0.009	0.007	0.222
**Male**												
Interaction term between P4P and period (reference group: comparison group in pre-P4P period)												
P4P group×Post-P4P 1 year	0.043	0.00	<0.001	0.028	0.009	0.003	0.006	0.00	0.402	0.017	0.007	0.011
P4P group×Post-P4P 2 year	0.050	0.00	<0.001	0.041	0.009	<0.001	-0.011	0.00	0.141	0.013	0.007	0.057
P4P group×Post-P4P 3 year	0.036	0.00	<0.001	0.031	0.010	0.001	-0.016	0.00	0.034	0.004	0.007	0.528

P4P—pay-for-performance program; HbA1c—glycated hemoglobin; LDL-C—l*ow*-density lipoprotein cholesterol; HDL-C—high-density lipoprotein cholesterol; TGs—triglycerides; ME—marginal effect; BSE—bootstrapped standard error.

All generalized estimating equations were controlled for sex, age groups, diabetes complication severity index score, Charlson comorbidity index score, hospitalization in the previous year, accreditation of hospitals and location of hospitals.

Table S3. Subgroup analysis for the number of chronic conditions

Characteristics	Incentivized items						Nonincentivized items					
	HbA1c (<7%)			LDL-C (<100 mg/dl)			HDL-C (>40 mg/dl)			TGs (<200 mg/dl)		
	ME	BSE	p-value	ME	BSE	p-value	ME	BSE	p-value	ME	BSE	p-value
Number of chronic conditions <2 (excluding diabetes)												
Interaction term between P4P and period (reference group: comparison group in pre-P4P period)												
P4P group×Post-P4P 1 year	0.032	0.01	0.001	0.041	0.01	<0.001	0.000	0.007	0.989	0.020	0.007	0.004
P4P group×Post-P4P 2 year	0.040	0.01	<0.001	0.048	0.01	<0.001	-0.011	0.007	0.122	0.022	0.007	0.003
P4P group×Post-P4P 3 year	0.023	0.01	0.021	0.056	0.01	<0.001	-0.013	0.007	0.090	0.021	0.007	0.006
Number of chronic conditions 2+ (excluding diabetes)												
Interaction term between P4P and period (reference group: comparison group in pre-P4P period)												
P4P group×Post-P4P 1 year	0.036	0.00	<0.001	0.038	0.01	<0.001	0.012	0.007	0.106	0.014	0.007	0.043
P4P group×Post-P4P 2 year	0.028	0.00	0.003	0.042	0.01	<0.001	0.003	0.007	0.675	0.012	0.007	0.109
P4P group×Post-P4P 3 year	0.032	0.00	0.001	0.032	0.01	0.001	0.001	0.007	0.910	-0.006	0.007	0.400

P4P—pay-for-performance program; HbA1c—glycated hemoglobin; LDL-C—low-density lipoprotein cholesterol; HDL-C—high-density lipoprotein cholesterol; TGs—triglycerides; ME—marginal effect; BSE—bootstrapped standard error.

All generalized estimating equations were controlled for sex, age groups, diabetes complication severity index score, Charlson comorbidity index score, hospitalization in the previous year, accreditation of hospitals and location of hospitals.

Table S4. Adjusted estimations of the effects of the P4P program on the incentivized and nonincentivized intermediate outcomes by using random intercept logit model

Characteristics	Incentivized items						Nonincentivized items					
	HbA1c (<7%)			LDL-C (<100 mg/dl)			HDL-C (>40 mg/dl)			TGs (<200 mg/dl)		
	ME	BSE	p-value	ME	BSE	p-value	ME	BSE	p-value	ME	BSE	p-value
P4P group (reference group: comparison group)	-0.097	0.005	<0.001	-0.008	0.006	0.171	0.013	0.003	<0.001	0.001	0.002	0.679
Period (reference group: pre-P4P period)												
Post-P4P 1 year	0.011	0.003	<0.001	0.044	0.004	<0.001	0.006	0.003	0.037	0.002	0.002	0.353
Post-P4P 2 year	-0.007	0.003	0.033	0.047	0.004	<0.001	0.016	0.003	<0.001	0.009	0.002	<0.001
Post-P4P 3 year	-0.003	0.003	0.410	0.084	0.004	<0.001	0.034	0.003	<0.001	0.020	0.002	<0.001
Interaction term between P4P and period (ref: comparison group in pre-P4P period)												
P4P group×Post-P4P 1 year	0.059	0.007	<0.001	0.039	0.008	<0.001	0.006	0.004	0.093	0.015	0.004	<0.001
P4P group×Post-P4P 2 year	0.066	0.006	<0.001	0.045	0.008	<0.001	-0.003	0.004	0.516	0.016	0.004	<0.001
P4P group×Post-P4P 3 year	0.066	0.007	<0.001	0.044	0.008	<0.001	-0.006	0.004	0.205	0.007	0.004	0.078
Male	-0.009	0.002	<0.001	0.044	0.002	<0.001	-0.211	0.001	<0.001	-0.025	0.001	<0.001
Age group (reference group: <60)												
60-74	0.060	0.003	<0.001	0.049	0.004	<0.001	0.035	0.003	<0.001	0.063	0.003	<0.001
75+	0.094	0.005	<0.001	0.102	0.005	<0.001	0.006	0.004	0.094	0.086	0.004	<0.001
DCSI score (reference group: Score 0)												
Score 1	-0.023	0.004	<0.001	0.039	0.004	<0.001	-0.010	0.003	0.001	-0.004	0.002	0.101
Score 2	-0.036	0.003	<0.001	0.059	0.004	<0.001	-0.018	0.003	<0.001	-0.003	0.002	0.145
CCI score (reference group: score 1)												
Score 2	0.017	0.003	<0.001	-0.002	0.003	0.472	-0.009	0.003	<0.001	0.002	0.002	0.303
Score 3+	0.032	0.005	<0.001	-0.006	0.005	0.252	-0.023	0.004	<0.001	-0.003	0.003	0.371
Hospitalization in the previous year (reference group: No)	0.014	0.003	<0.001	0.003	0.004	0.421	-0.007	0.003	0.007	-0.006	0.002	0.013
Medical center/Regional hospital (reference group: District hospital/clinics)												
Location of hospitals (reference group: Kao-ping and eastern regions)	0.011	0.003	<0.001	0.019	0.004	<0.001	-0.008	0.003	0.002	0.007	0.002	<0.001
Taipei and northern regions	0.001	0.003	0.815	-0.041	0.004	<0.001	0.003	0.003	0.293	-0.021	0.002	<0.001
Central and southern regions	-0.015	0.003	<0.001	-0.006	0.004	0.091	0.003	0.004	0.356	-0.005	0.002	0.027

P4P—pay-for-performance program; DCSI—Diabetes Complication Severity Index; CCI—Charlson Comorbidity Index; HbA1c—glycated hemoglobin; LDL-C—l*ow*-density lipoprotein cholesterol; HDL-C—high-density lipoprotein cholesterol; TGs—triglycerides; ME—marginal effect; BSE—bootstrapped standard error.

Table S4. Placebo differences-in-differences test before the enrollment process of the P4P program

	β	SE	p value
P4P group (reference group: comparison group)	-0.007	0.004	0.088
Period (reference group: pre-P4P 10 year)			
Pre-P4P 9 year	0.004	0.003	0.257
Pre-P4P 8 year	0.006	0.003	0.082
Pre-P4P 7 year	0.005	0.003	0.111
Pre-P4P 6 year	0.006	0.004	0.112
Pre-P4P 5 year	0.008	0.004	0.023
Pre-P4P 4 year	0.005	0.004	0.189
Pre-P4P 3 year	0.014	0.004	0.000
Pre-P4P 2 year	-0.003	0.004	0.498
Interaction term between P4P and period (reference group: comparison group in pre-P4P period)			
P4P group×Pre-P4P 9 year	-0.005	0.005	0.343
P4P group× Pre-P4P 8 year	-0.007	0.006	0.194
P4P group× Pre-P4P 7 year	-0.002	0.006	0.696
P4P group× Pre-P4P 6 year	-0.006	0.006	0.293
P4P group× Pre-P4P 5 year	-0.008	0.006	0.193
P4P group× Pre-P4P 4 year	0.000	0.006	0.948
P4P group× Pre-P4P 3 year	0.005	0.006	0.375
P4P group× Pre-P4P 2 year	0.019	0.006	0.002
Age groups (reference group: <60)			
60-74	-0.009	0.002	<0.001
75+	0.012	0.003	<0.001
Male	-0.004	0.002	0.024
DCSI score (reference group: score 0)			
Score 1	0.042	0.002	<0.001
Score 2	0.113	0.003	<0.001
CCI score (reference group: score 1)			
Score 2	0.052	0.002	<0.001
Score 3+	0.132	0.003	<0.001

P4P—pay-for-performance program; DCSI—Diabetes Complication Severity Index; CCI—Charlson Comorbidity Index
